# Combining ultraconserved elements and mtDNA data to uncover lineage diversity in a Mexican highland frog (*Sarcohyla*; Hylidae)

**DOI:** 10.7717/peerj.6045

**Published:** 2018-12-11

**Authors:** Eugenia Zarza, Elizabeth M. Connors, James M. Maley, Whitney L.E. Tsai, Peter Heimes, Moises Kaplan, John E. McCormack

**Affiliations:** 1 Moore Laboratory of Zoology, Occidental College, Los Angeles, CA, United States of America; 2 Mexico City, Mexico; 3 Ann Arbor, MI, United States of America

**Keywords:** Genomics, Population genetics, Phylogeography, Phylogenetics, Systematics, Species limits, Hylinae, Biodiversity, Mexico

## Abstract

Molecular studies have uncovered significant diversity in the Mexican Highlands, leading to the description of many new endemic species. DNA approaches to this kind of species discovery have included both mitochondrial DNA (mtDNA) sequencing and multilocus genomic methods. While these marker types have often been pitted against one another, there are benefits to deploying them together, as linked mtDNA data can provide the bridge between uncovering lineages through rigorous multilocus genomic analysis and identifying lineages through comparison to existing mtDNA databases. Here, we apply one class of multilocus genomic marker, ultraconserved elements (UCEs), and linked mtDNA data to a species complex of frogs (*Sarcohyla bistincta*, Hylidae) found in the Mexican Highlands. We generated data from 1,891 UCEs, which contained 1,742 informative SNPs for *S. bistincta* and closely related species and captured mitochondrial genomes for most samples. Genetic analyses based on both whole loci and SNPs agree there are six to seven distinct lineages within what is currently described as *S. bistincta*. Phylogenies from UCEs and mtDNA mostly agreed in their topologies, and the few differences suggested a more complex evolutionary history of the mtDNA marker. Our study demonstrates that the Mexican Highlands still hold substantial undescribed diversity, making their conservation a particularly urgent goal. The Trans-Mexican Volcanic Range stands out as a significant geographic feature in *Sarcohyla* and may have acted as a dispersal corridor for *S. bistincta* to spread to the north. Combining multilocus genomic data with linked mtDNA data is a useful approach for identifying potential new species and associating them with already described taxa, which will be especially important in groups with undescribed subadult phenotypes and cryptic species.

## Introduction

The Mexican Highlands are a global biodiversity hotspot (Myers et al., 2000). Recent molecular studies have uncovered significant diversity in the Mexican Highlands, leading to the description of new endemic species or the elevation of former subspecies to species rank (McCormack et al., 2008; Bryson Jr et al., 2010; Bryson et al., 2011; Bryson Jr, García-Vázquez & Riddle, 2012a; Bryson, García-Vázquez & Riddle, 2012b; Rovito et al., 2015; Campbell et al., 2018). At the same time, habitat loss threatens many of these species before they have even been described (Ponce-Reyes et al., 2012). Amphibians are particularly sensitive to habitat alterations, and many are threatened by habitat loss and invasive diseases (Stuart et al., 2008). Because most amphibians have reduced dispersal, they also show strong patterns of microendemism (Parra-Olea, Flores-Villela & Mendoza-Almeralla, 2014), meaning that important pockets of diversity and new species are still being uncovered in the Mexican Highlands (Meik et al., 2006; Campbell, Blancas-Hernández & Smith, 2009; Bryson Jr et al., 2018; Caviedes-Solis & Leaché, 2018).

The genus *Sarcohyla*, which is considered distinct from *Plectrohyla* by some authors to reflect those species west of the Isthmus of Tehuantepec (Duellman, Marion & Hedges, 2016), contains 24 species of stream-dwelling frogs, many of them critically endangered. Many species have never been seen after their original discovery (Stuart et al., 2008), and some are thought to be in serious decline or extinct (Lips et al., 2004). The actual number of species in the genus, their relationships, and geographic ranges are not well known (Duellman, 2001; Faivovich et al., 2009; Duellman, Marion & Hedges, 2016).

**Figure 1:**
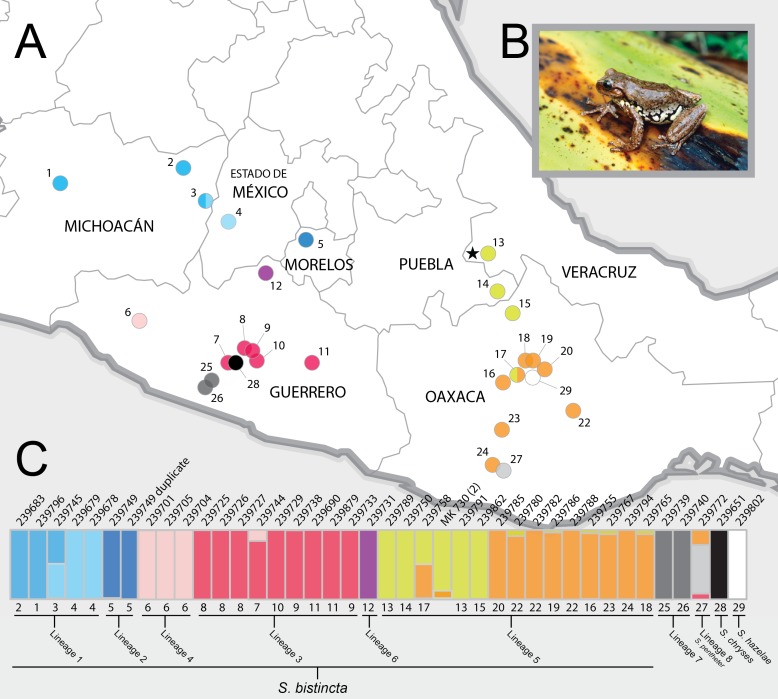
Sampling sites and Structure plots. (A) Map of central Mexico showing sampling sites for *S. bistincta* and close outgroups, with numbers matching localities listed in Table 1 and colors matching Structure results below. Unsampled parts of the distribution of *S. bistincta* are shown in Fig. S1. Star indicates the type locality; (B) *S. bistincta* individual from near site 1 (recently described as a new species, *S. hapsa*, see text); (C) Composite results of repeated Structure runs at *K* = 2 showing the finest detectable structure in the genetic data. Each vertical line represents an individual labeled with its UMMZ catalog number above and, the site number and the lineages indicated by the species delimitation SNAPP analysis. One tissue voucher MK 730 (2) could not be linked to a specimen voucher and thus its geographic locality is unknown. Photo credit: Peter Heimes.

The species *S. bistincta* is the most broadly distributed member of the genus (Figs. 1A, 1B). It occurs in several mountain ranges in central Mexico separated by lowland barriers and might therefore consist of multiple distinct lineages or even species. Studies of many broadly distributed highland species across this region have revealed cryptic lineages (McCormack et al., 2008; Bryson et al., 2011; Leaché et al., 2013; Navarro-Sigüenza, García-Hernández & Peterson, 2013; Mastretta-Yanes et al., 2015; Bryson Jr et al., 2017), some later described at the species level. Amphibians, it seems, are especially prone to under-splitting in the Mexican Highlands (Rovito et al., 2012; Rovito et al., 2013; Bryson Jr et al., 2018).

We assess lineage diversity in *S. bistincta* using multilocus genomic markers collected via next-generation sequencing (NGS) and mitochondrial DNA (mtDNA). Often, mtDNA and multilocus nuclear markers have been pitted against one another in biodiversity discovery (Moritz & Cicero, 2004; Zink & Barrowclough, 2008; Edwards & Bensch, 2009). Mitochondrial DNA offers many benefits, including economy, efficiency, and wide comparative potential across species and studies (Avise, 1998; Zink & Barrowclough, 2008). Consequently, there is now a vast trove of mtDNA sequences on GenBank. A drawback of using only mtDNA is that a single marker will often fail to accurately depict the speciation process (Moritz & Cicero, 2004; Edwards & Bensch, 2009). In response, multilocus methods have multiplied (Edwards, 2009; Fujita et al., 2012). While multiple loci help model a more realistic speciation process, multilocus studies suffer from the lack of a standardized marker set, which limits the ability to link uncovered lineages with species already identified and described in prior studies and in public databases (Coissac et al., 2016). This is especially important in groups with multiple subadult phenotypes (e.g., insects and frogs) and where adult phenotypes are conserved across species. While many studies have used both types of markers, it is increasingly uncommon in the genomic era to explore the benefits of linked mtDNA and NGS data at the level of the individual for lineage discovery and identification.

Our main goal was to use broad sampling within *S. bistincta* to uncover as many distinct genetic lineages as possible and determine their relationships to one another, which could inform later efforts to delimit them as species. We did not attempt to delimit species in the genetic sense because species delimitation and description is best done with integrative taxonomy using multiple sources of data in addition to DNA (Fujita et al., 2012; Leache et al., in press). For our markers, we used ultraconserved elements (UCEs). UCEs have been useful for deep-level systematics (Faircloth et al., 2012), but their utility at shallower scales around the species level is still coming into focus (Smith et al., 2014; McCormack, Tsai & Faircloth, 2016; Zarza et al., 2016). An added benefit of the UCE enrichment process (and all sequence capture methods) is that whole mtDNA genomes are often captured as off-target “bycatch” (Do Amaral et al., 2015), meaning individuals can end up with linked nuclear and mtDNA data (e.g., Zarza et al., 2016). Thus, our final goal was to determine if linked mtDNA data allowed for any further conclusions than those afforded by the UCEs alone.

## Methods

### Sampling and ingroup determination

We collected tadpoles from January to June 2004 across most of the known range of *Sarcohyla bistincta* (Duellman, 2001) in the Trans-Mexican Volcanic Belt of Michoacán, Morelos, and the state of México, the Sierra Madre del Sur of Guerrero, and the highlands of Oaxaca stretching into Puebla and Veracruz (Fig. 1; Table 1), including near the type locality in Veracruz (site 13 in Fig. 1). Tadpoles were targeted to improve sampling efficiency, which allowed for a larger sampling range and sample density. Collecting permits were issued by the Secretaría de Medio Ambiente y Recursos Naturales (permit #FAUT-0015). Unsampled parts of the *S. bistincta* range include the far west Trans-Mexican Belt in Michoacán and Jalisco, the far northwest in the Sierra Madre Occidental (Nayarit, Durango, and Sinaloa), and the far northeast in Hidalgo (see Fig. S1).

After collection from a sampling location with a dip net, tadpoles were, to the extent possible, separated by species based on morphology and reared to subadults in the laboratory prior to vouchering. This study complied with standard ethical guidelines for the rearing and collection of tadpoles. Species identification was based on the most recent diagnosis of *S. bistincta* and other closely related species (Duellman, 2001). One tadpole was chosen for the tissue voucher, while the other individuals became physical vouchers with museum catalog numbers. Thus, we provide both field numbers and catalog numbers in Table 1 to provide a link to both the exact genetic material (field number) and the associated phenotype voucher representing that genotype (museum catalog number). Before limiting our taxonomic sampling to 40 individuals including all known *S. bistincta* and closely related lineages, we ran preliminary phylogenetic analyses including broader sampling of 45 *Sarcohyla* individuals and an outgroup genus *Exerodonta* to ensure we had correctly identified the ingroup (Table S1; Fig. S2).

### Sequence capture and next-generation sequencing

We extracted genomic DNA from tissue using a Qiagen (Valencia, CA, USA) DNAeasy Blood and Tissue extraction kit. We visualized extractions on an agarose gel to ensure fragments were larger than 200 base pairs (bp) and quantified the resulting double-stranded DNA using a Qubit 2.0 Fluorometer (Carlsbad, CA, USA). For each sample, we sheared 100 µl of 20 ng/µl concentration DNA to a size distribution with its peak between 400 and 600 bp using a Bioruptor ultrasonicator (Diagenode, Liege, Belgium). We prepared libraries for each sheared sample with a KAPA (Boston, MA, USA) LTP library preparation kit for the Illumina platform, attaching custom indexing tags (Faircloth & Glenn, 2012) to each sample to allow sample pooling.

**Table 1:** Specimen information and summary statistics for *Sarcohyla bistincta* and closely related species.

Map Number^a^	Field Number^b^	Catalog Number^c^	Current Taxonomy	Lineage	State	Latitude	Longitude	mtDNA Accession
1	MK 618	UMMZ 239796	*S. hapsa*	1	Michoacán	19.7911	−100.6605	
2	MK 627-31	UMMZ 239683	*S. hapsa*	1	Michoacán	19.4266	−102.0736	
3	MK 666	UMMZ 239745	*S. hapsa*	1	Michoacán	19.3452	−100.3128	MH899567
4	MK 600	UMMZ 239679	*S. hapsa*	1	México	19.1501	−100.1469	MH899566
4	MK 600 (1)	UMMZ 239678	*S. hapsa*	1	México	19.1501	−100.1469	
5	MK 645	UMMZ 239749	*S. hapsa*	2	Morelos	18.9224	−99.2442	
6	MK 759	UMMZ 239701	*S. bistincta*	4	Guerrero	18.0013	−101.1716	MH899573
6	MK 760	UMMZ 239705	*S. bistincta*	4	Guerrero	18.0013	−101.1716	
6	MK 760 (2)	UMMZ 239704	*S. bistincta*	4	Guerrero	18.0013	−101.1716	
7	MK 691 (5)	UMMZ 239744	*S. bistincta*	3	Guerrero	17.5324	−99.8994	
8	MK 650 (1)	UMMZ 239725	*S. bistincta*	3	Guerrero	17.6843	−99.8034	
8	MK 650 (2)	UMMZ 239726	*S. bistincta*	3	Guerrero	17.6843	−99.8034	
8	MK 652	UMMZ 239727	*S. bistincta*	3	Guerrero	17.6843	−99.8034	MH899571
9	MK 671 (4)	UMMZ 239733	*S. bistincta*	3	Guerrero	17.6407	−99.6797	
9	MK 672	UMMZ 239738	*S. bistincta*	3	Guerrero	17.6407	−99.6797	
10	MK 656 (1)	UMMZ 239729	*S. bistincta*	3	Guerrero	17.5526	−99.6626	
11	MK 674 (1)	UMMZ 239690	*S. bistincta*	3	Guerrero	17.5087	−99.1258	
11	MK 675 (2)	UMMZ 239879	*S. bistincta*	3	Guerrero	17.5087	−99.1258	
12	MK 662	UMMZ 239731	*S. bistincta*	6	Guerrero	18.6359	−99.6491	MH899572
13	MK 697 (3)	UMMZ 239789	*S. bistincta*	5	Veracruz	18.6585	−97.1574	
13	MK 699 (1)	UMMZ 239791	*S. bistincta*	5	Veracruz	18.6477	−97.1574	
14	MK 700 (2)	UMMZ 239750	*S. bistincta*	5	Puebla	18.3220	−97.0285	
15	MK 705 (1)	UMMZ 239862	*S. bistincta*	5	Oaxaca	18.1576	−96.8684	
16	MK 715	UMMZ 239755	*S. bistincta*	5	Oaxaca	17.2390	−97.0032	
17	MK 716 (1)	UMMZ 239758	*S. bistincta*	5	Oaxaca	17.3036	−96.7930	
18	MK 718 (2)	UMMZ 239765	*S. bistincta*	5	Oaxaca	17.4211	−96.6876	
19	MK 755 (1)	UMMZ 239786	*S. bistincta*	5	Oaxaca	17.4153	−96.5671	
20	MK 751	UMMZ 239785	*S. bistincta*	5	Oaxaca	17.3160	−96.4435	MH899574
22	MK 748 (2)	UMMZ 239780	*S. bistincta*	5	Oaxaca	16.9791	−96.1364	
22	MK 748 (4)	UMMZ 239782	*S. bistincta*	5	Oaxaca	16.9791	−96.1364	
22	MK 767	UMMZ 239788	*S. bistincta*	5	Oaxaca	16.9859	−96.1358	
23	MK 721	UMMZ 239767	*S. bistincta*	5	Oaxaca	16.7377	−97.0384	
24	MK 766	UMMZ 239794	*S. bistincta*	5	Oaxaca	16.2522	−97.1536	
	MK 730 (2)	?	*S. bistincta*	5	?	?	?	
25	MK 685 (2)	UMMZ 239739	*S. bistincta*	7	Guerrero	17.3812	−100.2009	MH899575
26	MK 689 (2)	UMMZ 239740	*S. bistincta*	7	Guerrero	17.3000	−100.2792	
27	MK 727 (2)	UMMZ 239772	*S. pentheter*	8	Oaxaca	16.1916	−97.0958	MH899576
28	MK 691 (3)	UMMZ 239651	*S. chryses*	–	Guerrero	17.5324	−99.8994	MH899570
29	MK 770	UMMZ 239802	*S. hazelae*	–	Oaxaca	17.2216	−96.5839	MH899569

**Notes.**

a Map number in Fig. 1.

b The first three-digit number corresponds to a sampling location. If there is a second number in parentheses, this corresponds to different aquaria where tadpoles were sorted by species before rearing to subadults before vouchering.

c All specimens are from the University of Michigan Museum of Zoology. One specimen could not be linked to a catalog number.

We enriched pools of eight samples using a set of synthetic RNA probes that target 5,060 tetrapod UCEs (MYbaits_Tetrapods-UCE-5K kit, Mycroarray) following the standard UCE enrichment protocol (Faircloth & Glenn, 2012) with the following modification. Amphibians have large and variable genome sizes with a high percentage of repetitive DNA (Olmo, 1991). While we do not have information about the genome size and composition of *Sarcohyla* specifically, we wanted to decrease the potential risk of the probes hybridizing to repetitive elements (McCartney-Melstad, Mount & Shaffer, 2016). We thus increased by 6X the amount of the Cot-1 blocker, a synthetic DNA derived from chicken that binds to repetitive regions. After enrichment and recovery PCR, we verified the library size range with an Agilent 2100 Bioanalyzer (Palo Alto, CA, USA). We quantified the enriched pools using qPCR and combined them in equimolar ratios before sequencing on an Illumina HiSeq 2000 lane (100-bp paired-end cycle) at the University of California Santa Cruz Genome Technology Center.

### Bioinformatics of next-generation sequencing data

We demultiplexed the Illumina raw reads and converted them to FASTQ format with the program bcl2fastq v.1.8.4 (Illumina, Inc.). To eliminate adapter contamination and low quality bases, we trimmed the FASTQC output with illumiprocessor (Faircloth, 2013). We trimmed and assembled these reads into contigs with Trinity (Haas et al., 2013) and ABySS (Simpson et al., 2009), both of which are built into the PHYLUCE pipeline (Faircloth, 2015). PHYLUCE uses LASTZ (Harris, 2007) to align all assembled contigs to UCE probes in order to isolate only UCE contigs and to identify and eliminate paralogs.

### Phylogenetic trees from concatenated UCE data

We extracted UCE contigs into a single FASTA file and aligned the output for each locus using MAFFT (Katoh et al., 2002). We used a 75% threshold for missing data, meaning that 75% of the taxa needed to have data for a given locus for that locus to be included in the final concatenated matrix. Lower thresholds (i.e., more data) lead to diminishing returns (Hosner et al., 2015). We then constructed a maximum-likelihood (ML) tree in RAxML v8.0.19 (Stamatakis, 2014) under the GTRGAMMA model of evolution with 100 bootstrap searches, followed by a search for the tree with the highest likelihood.

Our more taxonomically inclusive data set with all available *Sarcohyla* and outgroup *Exerodonta xera* (Table S1) contained 1,866 UCE loci and 1,030,450 bp for a concatenated analysis. The resulting ML tree (Fig. S2) showed strong support for monophyly of the genus *Sarcohyla*, and identified *S. arborescandens* and *S. cyclada* as sister species that together form a clade sister to the rest of the *Sarcohyla* included in the study. We limited further analyses to a smaller data set of 40 samples (Table 1).

### Mitochondrial DNA assembly and analysis

We identified and assembled mtDNA genomes from off-target, trimmed Illumina reads using the reference genome of a closely related species, *Hyla annectans* Genbank accession number KM271781; (Ye et al., 2016). We used MITObim 1.7 (Hahn, Bachmann & Chevreux, 2013), a Perl wrapper for MIRA 4.0.2 (Chevreux, Wetter & Suhai, 1999), which takes an iterative mapping approach for assembly. We conducted *de novo* annotation of the assembled mtDNA regions with the MITOchondrial genome annotation Server, MITOS (Bernt et al., 2013). We selected for analysis only those individual genomes with MIRA quality score greater than 30. We aligned each protein-coding region separately in Geneious vR8 (Kearse et al., 2012) using MUSCLE (Edgar, 2004) and corrected the alignments manually when necessary and constructed a concatenated mtDNA matrix, which we also ran in RAxML v8.0.19.

We melded this mtDNA data with existing *Sarcohyla* and *Plectrohyla* mtDNA data on Genbank to determine whether any of the lineages we uncovered in *S. bistincta* relate to already-described species. We determined that *cytochrome b* is the best-represented mtDNA gene on Genbank for this group. We downloaded all existing *cytochrome b* sequences from *Sarcohyla* and *Plectrohyla* taxa. We combined these sequences with those from a subset of our *S. bistincta* samples, choosing the individual with the most raw reads from each major genetic lineage in the UCE tree. We aligned the trimmed, filtered reads for each individual to a *Sarcohyla cytochrome b* reference sequence and formed a consensus sequence for each individual from the mapped reads. We then created an alignment and generated a phylogeny using a Bayesian approach in BEAST v2.4.2 (Bouckaert et al., 2014). We repeated this process with 16S data because we suspected *S. pentheter* was closely related to our ingroup, but *S. pentheter* was not represented with *cyt b* sequence on Genbank.

### Calling SNPs from UCE loci

We called SNPs from UCE loci so that we could run genetic clustering tests and infer a species tree using SNAPP (Bryant et al., 2012), which uses SNPs as input data. Calling SNPs requires a reference sequence, and we chose the sample with the most UCE contigs recovered within the ingroup (UMMZ 239727). We then used BWA (Li & Durbin, 2009) to map the reads of each sample to this reference. We used SAMtools (Li et al., 2009) to sort the reads, and Picard (http://broadinstitute.github.io/picard) to identify and remove PCR duplicates. We realigned the mapped reads to minimize mismatched bases due to indels, and we removed indels using IndelRealigner in the Genome Analysis Toolkit 3.2, including (GATK; McKenna et al., 2010), as suggested by the Best Practices workflow (DePristo et al., 2011; Van der Auwera et al., 2013).

There is no SNP database available for this group, so we followed best practices for base recalibration for non-model organisms suggested by GATK (McKenna et al., 2010). This consists of (1) doing an initial round of calling SNPs on the original, uncalibrated data, (2) selecting the SNPs with the highest confidence (a minimum emission and call quality of 40 or more), and (3) using these SNPs as the reference database of known SNPs. We executed four rounds of base recalibration on the original data to filter out systematic error using a custom script. We called genotypes on the last recalibrated BAM file. We used vcf-tools (Danecek et al., 2011) to select one SNP per UCE and produce two data sets, one allowing 25% missing data (a conservative value) for STRUCTURE v 2.3.4 (Pritchard, Stephens & Donnelly, 2000), and one with no missing data—a requirement for SNAPP as implemented in BEAST v2.2.1 (Bouckaert et al., 2014).

### STRUCTURE analyses

We used STRUCTURE v2.3.4 (Pritchard, Stephens & Donnelly, 2000) as an unbiased way to explore the limits of fine-scale genetic structure in our data in order to compare this structure with lineages detected by phylogenetic analysis. Our intent with using STRUCTURE was not to determine the single most likely number of genetic clusters because it was not a goal of this manuscript to determine the number of species from genetic data alone. In the case of allopatric and closely related groups, it is best to use multiple sources of data in addition to genetics (e.g., phenotype and niche data) to conduct integrative taxonomy (Fujita et al., 2012). Our goal was to uncover as many distinct genetic lineages/groups as possible and determine their relationships to one another, which could inform later efforts to delimit them as species (e.g., Brown et al., 2007; Gowen et al., 2014). For these reasons, we did not use a method for identifying the “true K” (Evanno, Regnaut & Goudet, 2005), which can underestimate fine-scale population structure (Janes et al., 2017). We began by analyzing all individuals of *S. bistincta* plus two outgroup species *S. chryses* and *S. hazelae* under *K* = 4, reasoning that this would likely split out the two outgroups as well as reveal at most one division within *S. bistincta*. After this, each identified genetic cluster within *S. bistincta* was further analyzed at *K* = 2 until no coherent geographically-based structure was evident in the plots, as informed by prior studies (Brown et al., 2007; Gowen et al., 2014) and as recommended by the developer in the documentation for the program. In other words, we stopped analyzing when the addition of a new group led all individuals to be assigned roughly equally to that new group. All runs were completed twice and each used an admixture model and 10M generations with 1M generations as burn-in, which led to convergence for all analyses.

### Species tree from SNPs

We generated a species tree from the SNP matrix using SNAPP 1.1.10 (Bryant et al., 2012), a coalescent-based species tree analysis that uses SNPs an input data. This analysis included putative *S. bistincta* samples and close relatives, with *S. chryses* as the outgroup*.* For this run, we made no *a priori* assumptions about how individuals grouped into species and allowed each individual to be considered its own “species” (i.e., terminal tip) to allow the program to inform the number of lineages and in order to maximally visualize potential genetic connections among individuals in DensiTree v2.2.1 (Bouckaert et al., 2014). This kind of visualization of genetic connections is often itself revealing about the extent of gene flow among lineages (McCormack, Tsai & Faircloth, 2016; Zarza et al., 2016). We ran two instances of SNAPP for seven million generations, sampling every 100 steps, using default priors. We combined tree and parameter files from both runs with LogCombiner 2.1.3 and displayed the full set of likely species trees with Densitree v2.2.1.

## Results

### NGS summary statistics

Detailed summary statistics for each of the 40 individuals are described in Table S1. ABySS produced longer contigs than Trinity, and a higher number of UCE loci, so we used ABYSS contigs in all downstream analyses. Reads per sample ranged from 17,052 to 3,423,330 with an average of 1,185,165 reads. The number of identified UCEs ranged from 381 to 2,444 with an average of 1,976 UCEs. The mean length of individual UCE loci per individual ranged from 222 to 717 bp with an average of 522 bp. On average, 18% of the assembled contigs corresponded to unique UCE loci.

For SNP calling, across all samples, 9% of the trimmed reads mapped to our designated reference individual. SNP read depth ranged from 2.4 to 35.0 with an average depth of 21.2. The recalibration and quality control steps resulted in an initial matrix of 16,578 SNPs. After removing non-biallelic loci, selecting one SNP for every UCE, and allowing 25% missing data, there were 1,742 SNPs left in the STRUCTURE data set, while the 100% complete data matrix for SNAPP contained 399 SNPs.

### Structure analysis

As expected, the first run of STRUCTURE at *K* = 4 split the two outgroup species into distinct clusters and split the remaining individuals into two clusters. Further analysis of each cluster at *K* = 2 revealed 10 genetic clusters, identified with different colors and gray values in Fig. 1C (not including the white and black outgroups). There was a clear geographic component to population structure meaning that nearby individuals were assigned to similar clusters and most individuals did not share assignment among clusters, with the exception of two individuals (one in the Trans-Mexican Volcanic Belt and one in the Oaxacan Highlands) that showed evidence for connectivity among clusters.

### UCE phylogeny from concatenated data

The ML tree of the 40 samples was based on 1,891 UCE loci and 1,038,600 bp and recovered the presence of three main and well supported clades on relatively long branches, which corresponded to collections of genetic clusters from the Structure analysis: (1) a clade distributed across the Trans-Mexican Volcanic Belt (shaded blue in figures); (2) a clade inhabiting two disjunct areas along the coastal slopes of the Sierra Madre del Sur in Guerrero and Oaxaca (shaded gray); and (3) a clade broadly distributed in the Sierra Madre del Sur (shaded red and pink), the Oaxaca Highlands (shaded yellow and orange), and one individual in the southern portion of the Transvolcanic Belt (shaded purple). Further splitting within these clades was largely concordant with the Structure groups. The groups that showed evidence for genetic linkage in Structure with an admixed individual (e.g., yellow and orange) were likewise not monophyletic in the UCE tree.

One individual from Morelos (site 5) that nested within *S. bistincta* was labeled as a different species, *S. mykter*, a species that occurs in Guerrero. We suspect that this sample was mislabeled and is actually a duplicate of the *S. bistincta* sample already included in the study at site 5 because their field numbers are similar (last two digits transposed) and the two samples grouped together in all analyses. We have left this sample in all analyses, but have labeled it as a duplicate of *S. bistincta* UMMZ 239749. Another tissue voucher, MK 730 (2), could not be linked definitively to a physical voucher, and thus its geographic location is unknown. Its tip label has been left as the field number.

### mtDNA tree

**Figure 2:**
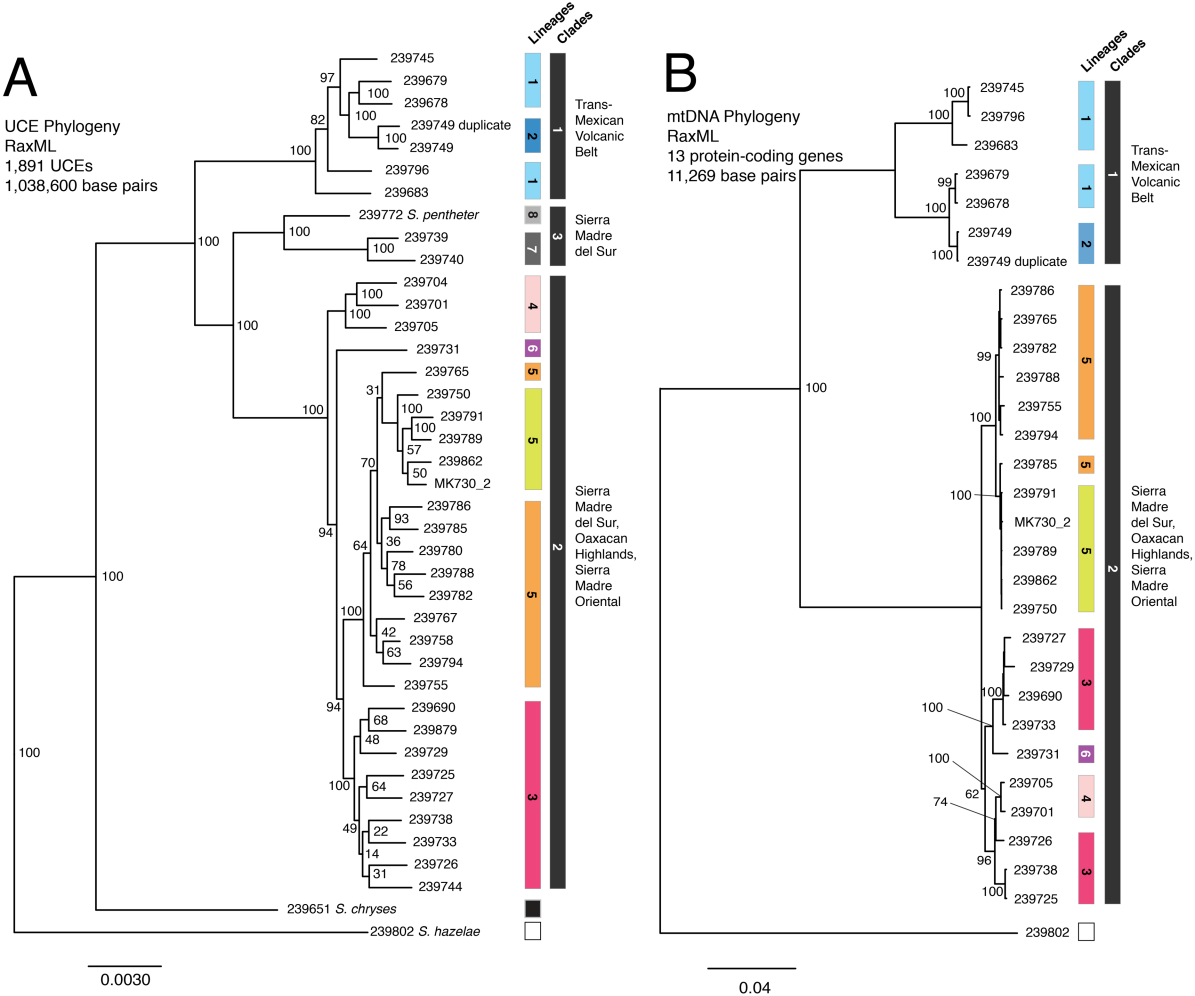
UCE and mtDNA phylogenies. (A) UCE tree; (B) mtDNA tree. Colors match Structure groups identified in Fig. 1. Tips are labeled with their UMMZ catalog number.

Our final concatenated mtDNA matrix was 11,269 base pairs including gaps, as coverage of the mtDNA genome varied from sample to sample in accordance with the non-targeted nature of the DNA collection (Table 1). Relationships in the ML tree (Fig. 2B) among the 29 (of the original 40) individuals with high quality scores were similar to the concatenated UCE tree with two key differences, both occurring within the large clade distributed across Guerrero and Oaxaca: (1) in the mtDNA tree, individuals from eastern and western Guerrero (shaded pink and red in figures) formed a clade, whereas they were more distantly related in the UCE tree; (2) in the mtDNA tree, individual UMMZ 239731 (shaded purple) was nested within the Guerrero (red/pink) clade instead of being sister to a much more inclusive clade, as in the UCE tree.

### SNAPP species tree

**Figure 3:**
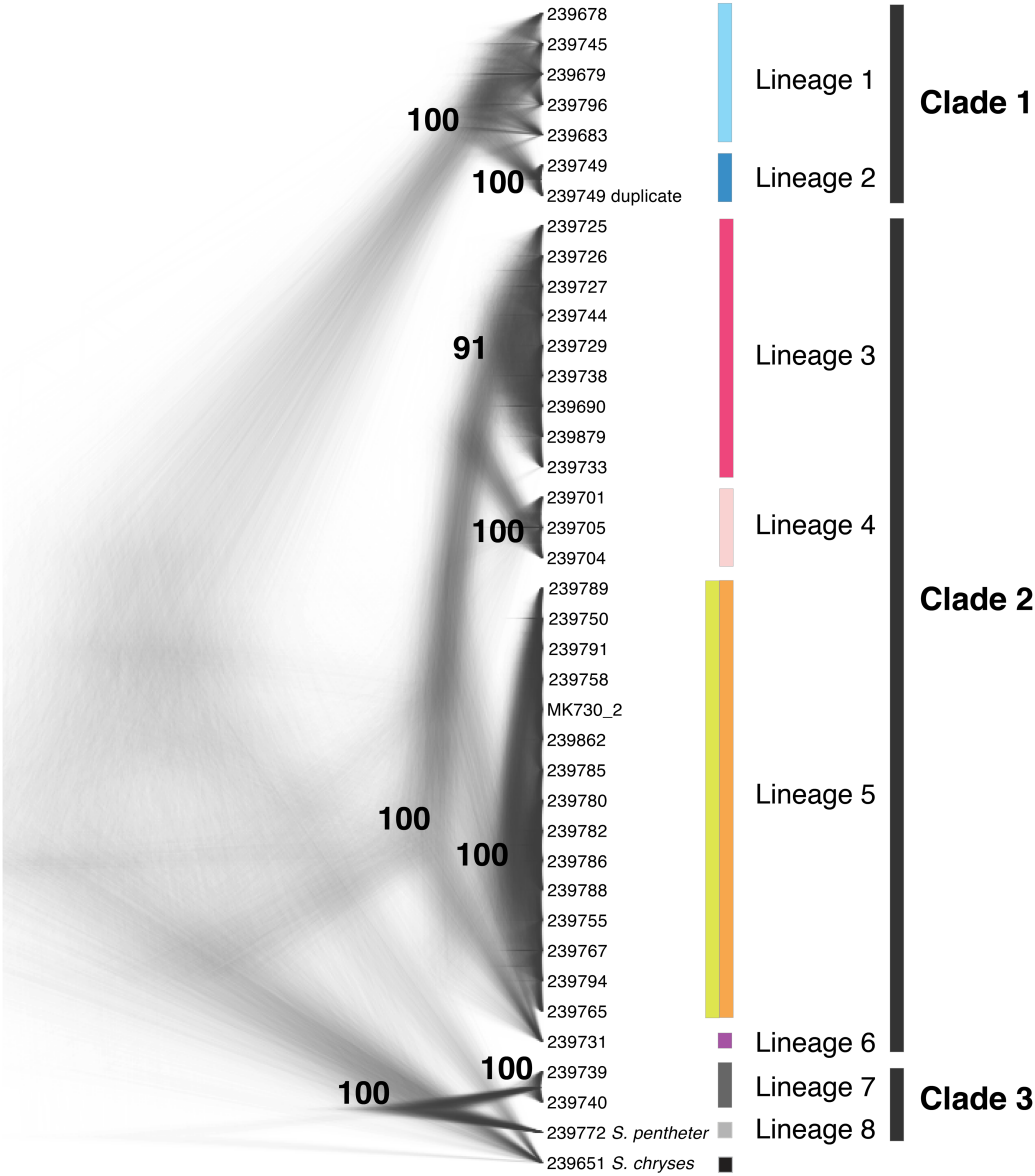
Species tree cloudogram. Cloudogram of the posterior distribution of SNAPP trees from 399 high-quality SNPs mined from UCE loci. Tip labels are UMMZ catalog numbers. Lineages and clades are discussed in text. Colors match genetic clusters from Fig. 1.

The SNAPP tree and its cloudogram of posterior species trees (Fig. 3) revealed eight well-supported lineages largely consistent with the genetic clusters in the Structure analysis (though not as finely split) and with relationships in the UCE and mtDNA gene trees. Where UCE and mtDNA data disagreed, the SNAPP species tree supported elements of both. For instance, the species tree agreed with the mtDNA gene tree, and not the UCE gene tree, that the eastern and western Guerrero individuals (red and pink groups) form a clade. In contrast, the species tree agreed with the UCE tree, and not the mtDNA tree, in the placement of individual UMMZ 239731 (purple group) not nested within other lineages.

### mtDNA phylogeny combining new data with Genbank sequences

Using 16S sequences, we determined that one lineage (UMMZ 239772) from Fig. 3 matched an *S. pentheter* sequence on Genbank. This individual had one of the lowest read counts of any sample and very few mtDNA reads. However, five Illumina reads mapped to 16S covering 421 bp of the 681 bp reference sequence (Genbank *S. pentheter* accession number DQ055825). Over this stretch, UMMZ 239772 was identical to the *S. pentheter* reference. As a point of comparison, UMMZ 239679 (a member of the blue *S. bistincta* Lineage 1 in the Transvolcanic Belt) had 70 differences across the 681 bp (10.3% divergence). This DNA identification of UMMZ 239772 as *S. pentheter* was later confirmed by re-examining the subadult specimen.

**Figure 4:**
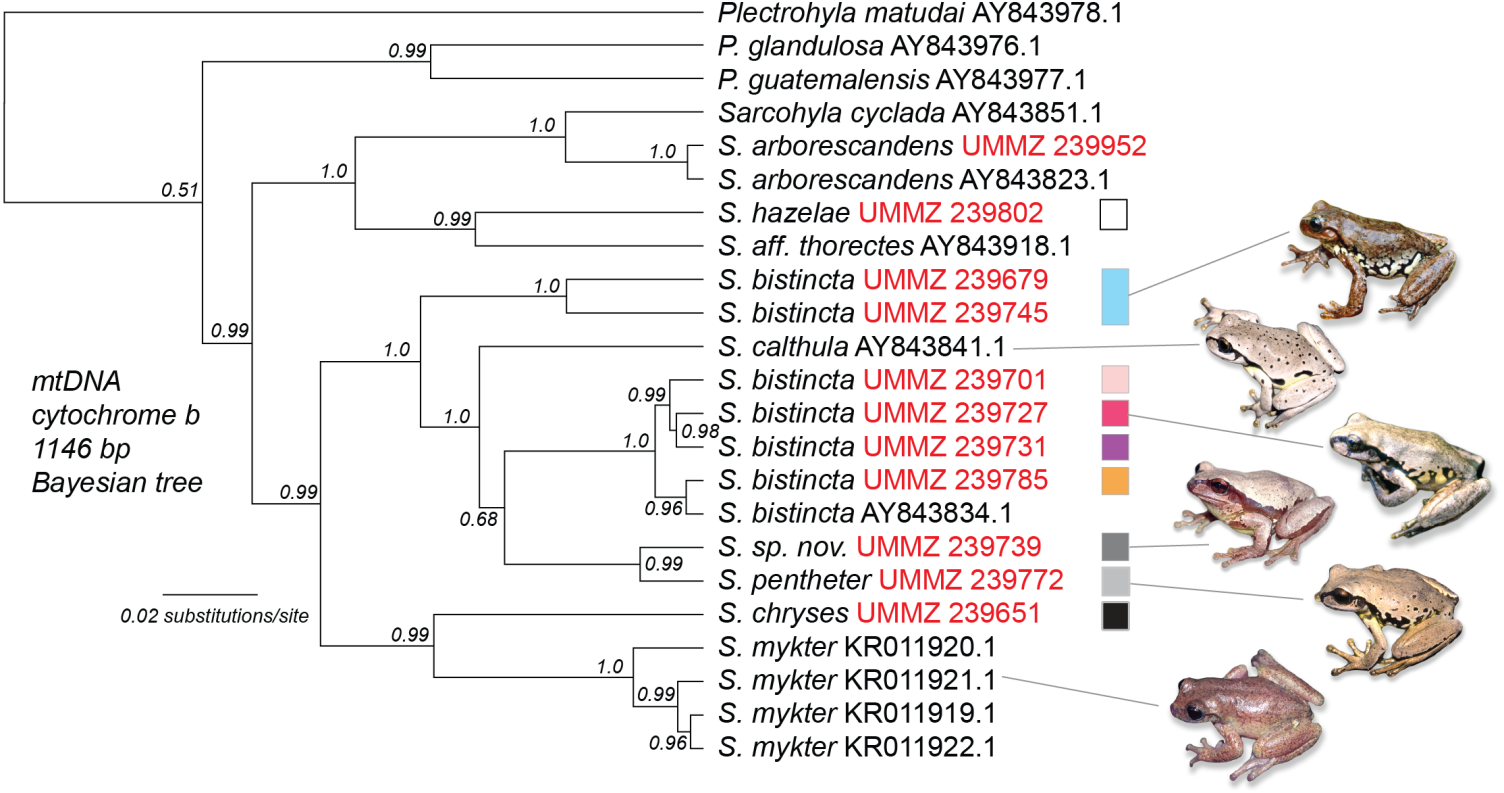
Combined Genbank phylogeny. Phylogeny of mtDNA for a subset of the individuals from this study (labeled with red UMMZ catalog numbers) combined with existing sequences from Genbank (labeled with accession numbers). Colored boxes relate to genetic lineages in prior figures. The tree was rooted with *Exerodonta xera*. Photo credit: Peter Heimes.

After confirming UMMZ 239679 as *S. pentheter*, we generated a Bayesian tree of *cytochrome b* combining the samples from this study with Genbank sequences (Fig. 4). This tree revealed not only that *S. pentheter* was nested within *S. bistincta*, but so was another species not included in our sampling, *S. calthula*. The tree also helped clarify relationships outside of *S. bistincta* by supporting *S. chryses* + *S. mykter* to be sister to the *S. bistincta* + *S. pentheter* + *S. calthula* clade.

## Discussion

### Bridging genomic data and mtDNA data for cryptic lineage discovery

Finding and identifying lineage diversity in broadly distributed species is an important area of study in systematics because terminal taxa (whether they are called species or not) are the fundamental unit of analysis in studies of biogeography, ecology, evolution, and conservation (Barrowclough et al., 2016). In this study, multilocus genomic data identified six to seven divergent lineages lumped under the name *Sarcohyla bistincta* (one of these was described as a new species while this manuscript was in review). Mitochondrial DNA data helped link our genomic data with existing databases to identify two already-described species as nesting within a paraphyletic *S. bistincta*.

The approach of discovering lineages with multilocus data and querying them against existing databases with linked mtDNA data should be especially useful in understudied groups where basic natural history information is lacking. While there is considerable debate around the choice of molecular markers in taxonomy, as highlighted by the ongoing conversation over DNA barcoding (Moritz & Cicero, 2004; DeSalle, Egan & Siddall, 2005; Ebach & Holdrege, 2005), our results show that genomic data and mtDNA data can be complementary, offering benefits that offset each other’s drawbacks (Rubinoff & Holland, 2005). In this case, UCE data provided a genomic portrait of divergence allowing for more robust conclusions about gene flow or its absence (see below) as compared to mtDNA data alone. Meanwhile, mtDNA data offset a drawback of UCEs that they are not well represented in existing genetic databases, and provided a bridge to prior studies and a vast database of sequences backed by vouchered specimens.

### Genetic lineage discovery in *Sarcohyla*

Our genomic results from different analyses were in broad agreement that there are at least six and perhaps seven distinct lineages among populations currently described as *S. bistincta*. Additionally, one already-described species appears to be nested within a broader clade that includes all currently described *S. bistincta* lineages, meaning that *S. bistincta* is paraphyletic. We use the species tree from Fig. 3 as a framework to outline lineage diversity in this group because this analysis, while based on a fraction of the data, deploys the most rigorous theoretical framework (the coalescent) and appears to represent a conservative blend of results from all analyses. Within this framework of three clades and eight lineages we discuss further genetic structure suggested by the gene trees and Structure results as well as discrepancies. We synthesize these results with three studies (Campbell et al., 2018; Caviedes-Solis & Nieto-Montes, 2018; Caviedes-Solis & Leaché, 2018), which were published after our work appeared in preprint form and while it was in peer review.

**Clade 1**—Trans-Mexican Volcanic Belt of central Mexico. This clade is sister to the rest of *S. bistincta* plus *S. pentheter* and is 10% divergent in mtDNA from other *S. bistincta*. This lineage was recently described as a new species, *S. hapsa* (Campbell et al., 2018). This clade might itself contain more than one species in the form of Lineages 1 and 2 below. Populations of *S. bistincta* in the Sierra Madre Occidental (Fig. S1), unsampled in our study, appear to fall within this clade (Caviedes-Solis & Leaché, 2018). Another genomic study suggested *S. calthula* is sister to this clade (Caviedes-Solis & Leaché, 2018). Our results based on much less data (Fig. 4) place *S. calthula* in a different location, sister to all other *S. bistincta* + *S. pentheter*.

Lineage 1 (light blue in Fig. 1)—Michoacán to western Mexico state. The Structure results show fine-scale genetic structure across this range. The presence of a geographic and genetic intermediate, as well as paraphyly of this lineage in the UCE and mtDNA gene trees, hints at continuity of gene flow along the distribution from sites 1 to 4 (Fig. 1). In addition to the unsampled populations mentioned above, some populations in far western Michoacán (Fig. S1) are also unsampled and could reveal further genetic structure.

Lineage 2 (dark blue in Fig. 1)—Morelos. Denser sampling between sites 4 and 5 could help determine whether the genetic distinctness of this individual in the mtDNA and UCE trees is a true discontinuity or the result of a sampling gap. This same sample was included in another genomic study of this group and was not noted as particularly distinctive (Caviedes-Solis & Leaché, 2018), but it does not appear that this study interrogated Clade 1 strongly for fine-scale structure.

**Clade 2**—Guerrero to Puebla and Veracruz and south through Oaxaca. This clade, which includes localities near the type specimen of *S. bistincta*, was also found to be monophyletic in two other studies (Caviedes-Solis & Nieto-Montes, 2018; Caviedes-Solis & Leaché, 2018).

Lineage 3 (red in Fig. 1)—Central and eastern Guerrero. Members of this lineage are distinct from Lineage 4 and are monophyletic in the UCE-based trees (both gene tree and species tree), but not in the mtDNA tree. Further sampling in between site 6 and site 7 would clarify whether the genetic discontinuity between Lineages 3 and 4 results from a sampling gap. Lineages 3 and 4 were not found to be distinct from one another in another genomic study (Caviedes-Solis & Leaché, 2018).

Lineage 4 (pink in Fig. 1)—Western Guerrero. This lineage is monophyletic in all trees, although only a few individuals were sampled from a single locality.

Lineage 5 (orange and yellow in Fig. 1)—Puebla, Veracruz, and Oaxaca. This lineage likely contains the type locality for *S. bistincta* (black star in Fig. 1A). Central and southern Oaxaca individuals (orange) are genetically distinct from individuals to the north (yellow). One genetic intermediate in central Oaxaca suggests genetic continuity across this range. An unsampled northern population in Hidalgo is most likely related to this lineage, and should be included in future studies. Another genomic study found even more fine-scale population structure across the range of this lineage (Caviedes-Solis & Leaché, 2018).

Lineage 6 (purple in Fig. 1)—far northern Guerrero. This lineage is distinct but represented by only a single individual. Its exact placement within Clade 2 varies depending on the analysis. Sampling more individuals is needed to determine how distinct this lineage might be and where it falls in the phylogeny. It was not noted as distinct in another genomic study (Caviedes-Solis & Leaché, 2018).

**Clade 3**—Pacific slope of Guerrero and Oaxaca. This clade contains two lineages, one of which is already described as a distinct species (*S. pentheter*). This clade was strongly supported in the UCE-based analyses, but we did not obtain mtDNA data from these samples.

Lineage 7 (dark gray in Fig. 1)—Pacific slope of Guerrero.

Lineage 8 (light gray in Fig. 1)—Pacific slope of Oaxaca. *S. pentheter*.

### Implications for the biogeography of the Mexican highlands

One biogeographic conclusion we can draw from the uncovered cryptic lineages is that the Trans-Mexican Volcanic Belt of central Mexico appears to have played an important role in generating some of the oldest diversity within this group. A rigorous biogeographic analysis complete with divergence dating was beyond the scope of our study, but it appears possible, given the 10% mtDNA sequence divergence, that the split of the lineage found in the Trans-Mexican Volcanic Belt—newly described as *S. hapsa* (Campbell et al., 2018)—dates to the final uplift of that range 2.5 million years ago (Ferrari et al., 1999). However, if *S. calthula* is sister to *S. hapsa*, as seems likely given the strong support for this relationship in Caviedes-Solis & Leaché (2018), then the divergence of *S. hapsa* would be more recent and more likely the result of dispersal to the Trans-Mexican Volcanic Belt during glacial periods. While firm conclusions must await rigorous analysis, the Trans-Mexican Volcanic Belt might have provided a bridge for the dispersal of *Sarcohyla* populations from strongholds in southeastern Mexico to the north into the Sierra Madre Occidental, similar to the biogeographic history of salamanders in the *Isthmura bellii* group (Bryson Jr et al., 2018). Regardless, our results add to the importance of the Trans-Mexican Volcanic Belt as a diversifying feature of Mexico’s highland flora and fauna (McCormack et al., 2008; Bryson Jr, García-Vázquez & Riddle, 2012a; Bryson, García-Vázquez & Riddle, 2012b; Ruiz-Sanchez & Specht, 2013; Mastretta-Yanes et al., 2015).

Another biogeographic pattern that is evident, though not quite as strongly supported, in our results is divergence between portions of the Sierra Madre del Sur in Guerrero and southern Oaxaca. Often considered part of one continuous landmass, this ancient mountain range is interrupted by the Sierra de Mixteca, which appears to have disrupted the genetic continuity of numerous species in this area (Nieto-Samaniego et al., 2006). Both our results, and those from Caviedes-Solis & Leaché (2018) support divergent genetic groups in the Guerrero and Oaxaca portions of the Sierra Madre del Sur, with the latter populations of *S. bistincta* showing closer affinity to the Oaxacan Highlands. The species tree and Structure results (both SNP-based analyses) showed a clearer pattern of differentiation between these two Sierra Madre del Sur regions, whereas reciprocal monophyly was lacking in the UCE and mtDNA phylogenies. Along with results showing high levels of genetic structure in the Oaxacan Highlands and at their interface with the Sierra Madre Oriental (Caviedes-Solis & Leaché, 2018), these results demonstrate how the parceling of the topography of central Mexico into “mountain ranges” belies a more complex history of their formation and impact on biodiversity.

### Discord between nuclear and mtDNA trees

With increasing genomic coverage, researchers are finding that gene flow is more common than previously thought (Mallet, Besansky & Hahn, 2016) and different DNA markers are often in conflict (Toews & Brelsford, 2012). On the whole, results from the nuclear genome and mtDNA, while they did not always perfectly support one another, were also not in strong conflict. There was one exception to this pattern that is worth discussing in detail, concerning Lineages 3, 4, and 6 in Clade 2 in Guerrero state (red, pink, and purple lineages in the figures). These three lineages were distinct from one another and each was monophyletic in the UCE phylogeny and species tree, both based on nuclear DNA. However, in the mtDNA tree, Lineage 3 was strongly supported as paraphyletic, with Lineages 4 and 6 nested within its limits.

To uncover the source of the discrepancy with any confidence would require, at minimum, more thorough geographic sampling. But the conflict between nuclear and mtDNA trees, in combination with the close geographic proximity of these lineages, suggests a role for gene flow in generating these discordant marker histories. Gene flow can create reticulate histories through issues with mitonuclear incompatibility (Hill & Johnson, 2013) or through the wholesale capture of the mitochondrial genome of one species by another (e.g., Bryson Jr et al., 2010; Bryson Jr et al., 2014). This possibility of gene flow signifies that lineages 3–6 (Clade 2), which contains samples near the type locality, should be grouped together as *S. bistincta* sensu stricto.

### Insights into broader *Sarcohyla* taxonomy and systematics

Additional insights into broader *Sarcohyla* relationships offered by the mtDNA tree include support for a previously hypothesized close relationship between *S. hazelae* and *S. thorectes* (Faivovich et al., 2009; Caviedes-Solis & Nieto-Montes, 2018), and a sister relationship between *S. mykter* and *S. chryses* (also found by Caviedes-Solis & Leaché (2018)). As *Sarcohyla* is very poorly represented by voucher specimens and DNA sequences (Fig. S1), a complete understanding of the history of this genus must await more complete taxonomic and genomic sampling. However, if recent studies like this one on *S. bistincta* and close relatives are any indication, the genus as a whole likely harbors significant undescribed diversity at the species level.

These studies of diversity within *Sarcohyla* come at a time when frogs and amphibians are experiencing global declines (Stuart et al., 2004). Surveys efforts suggest that some *Sarcohyla* species, especially those in the Oaxacan Highlands, might have gone extinct before they could ever be well studied (Lips et al., 2004), and existing species ranges do not overlap well with existing conservation areas (Caviedes-Solis & Leaché, 2018). Although recent resurveys provide hope for rediscovery of some *Sarcohyla* species (Delia, Whitney & Burkhardt, 2013), the protection of Mesoamerica’s cloud forests is imperative for their continuing survival.

### UCEs as a universal genomic marker set for species discovery?

Although UCEs are currently not well represented in genetic databases, as their use grows it is worth considering whether they might answer the call for an multilocus DNA marker set that satisfies criteria for use in species discovery (so-called “extended DNA barcodes” sensu (Coissac et al., 2016)): ease-of-use, universality, and genomic coverage. UCE probe sets are now available for many taxonomic groups (Faircloth et al., 2013; Faircloth et al., 2015; Starrett et al., 2016). They capture a discrete and replicable portion of the genome, in this case a set of around 2,000 loci that queries approximately 1,000,000 base pairs, or 0.02% of the frog genome. The replicable nature of UCEs sets them apart from other types of nuclear genomic markers, like RAD loci, which can vary from experiment to experiment (DaCosta & Sorenson, 2014). Other alternatives for “extended DNA barcodes” exist, like exons, but in mammals exons had fewer loci conserved over broad taxonomic scales, making them less able to be universally applied (McCormack et al., 2012). Few studies have applied UCEs to fine-scale population structure, for example, species delimitation (but see Oswald et al., 2016). While we did not attempt to delimit species here, a future research avenue could be to determine how much locus-sharing occurs among studies and species, and whether objective benchmarks exist to identify candidate species.

## Conclusions

This study shows there is still substantial diversity remaining to be described in the Mexican Highlands. Genetic studies to uncover this diversity might use different approaches and marker types, but these efforts need not be in opposition. As our study shows, NGS and mtDNA data work well together, and *Sarcohyla* lineages uncovered via multilocus methods could be checked against mtDNA databases to match the uncovered lineages with potential existing species. We expect this framework will be especially useful for species with undescribed lineage diversity and species with undescribed larval stages. We found that *S. bistincta* is not only paraphyletic, but also contains lineages that might meet criteria for species status. As the destruction of native habitats continues apace, it is important that we identify distinctive lineages and geographic centers of diversity before they are lost.

##  Supplemental Information

10.7717/peerj.6045/supp-1 Supplemental Information 1 Supplemental Table and Figures Table S1. Information and summary statistics on all 45 samples used to determine the ingroup for this study.
Fig. S1. Sampled and unsampled parts of *S. bistincta* range in relation to known distributions (or localities, where distributional information is lacking) of other *Sarcohyla* species.
Fig. S2. UCE tree of 45 samples of *Sarcohyla* and outgroup *Exerodonta xera* used to determine the ingroup.

## Abbreviations

UCEs: ultraconserved elements
SNPs: single nucleotide polymorphisms
mtDNA: mitochondrial DNA
bp: base pairs
ML: maximum-likelihood
UMMZ: University of Michigan Museum of Zoology
RAD loci: restriction digest-associated loci

## Additional Information and Declarations

### Field Study Permissions

The following information was supplied relating to field study approvals (i.e., approving body and any reference numbers): Collecting permits were issued by the Secretaría de Medio Ambiente y Recursos Naturales (permit #FAUT-0015).

### DNA Deposition

The following information was supplied regarding the deposition of DNA sequences: The datasets generated and analyzed in the current study are available on Genbank (BioProject ID PRJNA393258).

### Data Availability

The following information was supplied regarding data availability: The datasets generated and analyzed in the current study are available on Genbank (UCE data: BioProject ID PRJNA393258; mtDNA data: MH899566–MH899576).
