# Supplementary material for: Combining ultraconserved elements and mtDNA data to uncover lineage diversity in a Mexican highland frog (Sarcohyla; Hylidae)
Source: PeerJ. 2018 Dec 11;6:e6045. doi: 10.7717/peerj.6045 (PMC6294053; doi:10.7717/peerj.6045)

Table S1. Information and summary statistics on all 45 samples used to determine the ingroup for this study.

| Field Number | UMMZ Number | Current Taxonomy | State | Locality | Latitude | Longitude | Trimmed Paired Reads | UCEs | Average UCE length | mtDNA reads | mtDNA average coverage | mtDNA average quality | Fig. 1 Map Number | mtDNA Accession |
|---|---|---|---|---|---|---|---|---|---|---|---|---|---|---|
| MK 618 | UMMZ 239796 | *Sarcohyla bistincta* | MICHOACAN | LOS AZUFRES / SAN PEDRO ROAD | 19.791051 | -100.660542 | 1,889,670 | 2323 | 529.7 | 3381 | 20.8 | 86 | 1 | |
| MK 627-31 | UMMZ 239683 | *Sarcohyla bistincta* | MICHOACAN | PARQUE BARRANCA DEL CUPATITZIO URUAPAN | 19.426646 | -102.073574 | 1,933,019 | 2254 | 370.9 | 3415 | 20.8 | 85 | 2 | |
| MK 666 | UMMZ 239745 | *Sarcohyla bistincta* | MICHOACAN | 13 KMS ROAD ZITACUARO MACHERO (LITTLE TOWN WERE THERE IS A MONARCH BUTTERFLY SANCTUARY) | 19.3452 | -100.3128 | 2,186,871 | 2350 | 621.3 | 3900 | 23.4 | 83 | 3 | MH899567 |
| MK 600 | UMMZ 239679 | *Sarcohyla bistincta* | MEXICO | CARRETERA VALLE DE BRAVO-SAN PEDRO TENEYAC ARROYO 1 (6860 FEET) (POR ALBARRADA) BOSQUE BIEN CONSERVADO Y HUMEDO | 19.150083 | -100.1469 | 932,448 | 2174 | 500.8 | 2377 | 15.7 | 83 | 4 | MH899566 |
| MK 600 (1) | UMMZ 239678 | *Sarcohyla bistincta* | MEXICO | CARRETERA VALLE DE BRAVO-SAN PEDRO TENEYAC ARROYO 1 (6860 FEET) (POR ALBARRADA) BOSQUE BIEN CONSERVADO Y HUMEDO | 19.150083 | -100.1469 | 1,217,987 | 2267 | 532.8 | 2570 | 16.4 | 84 | 4 | |
| MK 645 | UMMZ 239749 | *Sarcohyla bistincta* | MORELOS | 2 KMS OUT OF CUERNAVACA ON THE CUERNAVACA CHALMA RD. (PASSING CONJUNTO CERRADO) OF LA BARRANCA WHERE THE SALTO SAN ANTONIO IS | 18.922402 | -99.244151 | 2,217,054 | 2406 | 532.5 | 3494 | 21.8 | 86 | 5 | |
| MK 645 dupe | UMMZ 239749 | *Sarcohyla bistincta* | MORELOS | 2 KMS OUT OF CUERNAVACA ON THE CUERNAVACA CHALMA RD. (PASSING CONJUNTO CERRADO) OF LA BARRANCA WHERE THE SALTO SAN ANTONIO IS | 18.922402 | -99.244151 | 548,585 | 1964 | 513.0 | 841 | 7.4 | 60 | 5 | |
| MK 759 | UMMZ 239701 | *Sarcohyla bistincta* | GUERRERO | CA 100 DEG KMS FROM CIUDAD ALTAMIRANO VIA IXTAPA ZIHUATANEJO | 18.0013 | -101.1716 | 1,224,835 | 2199 | 557.6 | 2522 | 16.2 | 84 | 6 | MH899573 |
| MK 760 | UMMZ 239705 | *Sarcohyla bistincta* | GUERRERO | CA 100 DEG KMS FROM CIUDAD ALTAMIRANO VIA IXTAPA ZIHUATANEJO | 18.0013 | -101.1716 | 1,074,315 | 2203 | 559.4 | 743 | 6.9 | 58 | 6 | |
| MK 760 (2) | UMMZ 239704 | *Sarcohyla bistincta* | GUERRERO | CA 100 DEG KMS FROM CIUDAD ALTAMIRANO VIA IXTAPA ZIHUATANEJO | 18.0013 | -101.1716 | 927,774 | 2246 | 579.2 | 906 | 9.4 | 6 | 6 | |
| MK 691 (5) | UMMZ 239744 | *Sarcohyla bistincta* | GUERRERO | 2-3 KMS IN THE ROAD TO JALEACA FROM POINT WHERE ROAD TRIFURCATE TO PTO. DEL GALLO / YEXTLA / ANDJALEACA.COMMING FROM CARRIZAL DE BRAVO / IN RIVER UNDER THE BRIDGE | 17.5324 | -99.8994 | 2,084,203 | 2353 | 672.5 | 1678 | 11.9 | 80 | 7 | |
| MK 650 (1) | UMMZ 239725 | *Sarcohyla bistincta* | GUERRERO | MOUNTAINS W. OF CHILPANCINGO / TOWN LOS MORROS | 17.6843 | -99.80339 | 941,101 | 2124 | 556.6 | 1278 | 9.7 | 74 | 8 | |
| MK 650 (2) | UMMZ 239726 | *Sarcohyla bistincta* | GUERRERO | MOUNTAINS W. OF CHILPANCINGO / TOWN LOS MORROS | 17.6843 | -99.80339 | 2,224,898 | 2394 | 506.4 | 1737 | 12.2 | 79 | 8 | |
| MK 652 | UMMZ 239727 | *Sarcohyla bistincta* | GUERRERO | MOUNTAINS W. OF CHILPANCINGO / TOWN LOS MORROS | 17.6843 | -99.80339 | 3,423,330 | 2444 | 526.0 | 1396 | 10.4 | 74 | 8 | MH899571 |
| MK 671 (4) | UMMZ 239733 | *Sarcohyla bistincta* | GUERRERO | BEHIND CHICHIHUALCO / ON ROAD TO CARRIZAL DE BRAVO / 2 KMS FROM ENTRONQUE | 17.6407 | -99.6797 | 1,012,300 | 2107 | 557.0 | 842 | 7.4 | 62 | 9 | |
| MK 672 | UMMZ 239738 | *Sarcohyla bistincta* | GUERRERO | BEHIND CHICHIHUALCO / ON ROAD TO CARRIZAL DE BRAVO / 2 KMS FROM ENTRONQUE | 17.6407 | -99.6797 | 297,782 | 1667 | 439.8 | 400 | 5.1 | 41 | 9 | |
| MK 656 (1) | UMMZ 239729 | *Sarcohyla bistincta* | GUERRERO | 3 KMS FROM THE TOWN OF OMILTEMI IN THE ROAD OMILTEMI CHILPANCINGO | 17.552603 | -99.662569 | 580,194 | 1950 | 497.6 | 547 | 5.9 | 42 | 10 | |
| MK 674 (1) | UMMZ 239690 | *Sarcohyla bistincta* | GUERRERO | ON ATZACUALOYA HUEYCATENANGO RD. | 17.5087 | -99.1258 | 659,418 | 1941 | 565.4 | 1086 | 8.7 | 68 | 11 | |
| MK 675 (2) | UMMZ 239879 | *Sarcohyla bistincta* | GUERRERO | ON ATZACUALOYA HUEYCATENANGO RD. | 17.5087 | -99.1258 | 336,474 | 1565 | 519.1 | 227 | 4.2 | 24 | 11 | |
| MK 662 | UMMZ 239731 | *Sarcohyla bistincta* | GUERRERO | 1.6 KMS FROM THE TOWN OF TETIPAC ON THE TETIPAC TAXCO ROAD (MAYBE ARROYO LAS DAMAS) | 18.635895 | -99.6491 | 1,137,742 | 2130 | 548.7 | 796 | 7.2 | 56 | 12 | MH899572 |
| MK 697 (3) | UMMZ 239789 | *Sarcohyla bistincta* | VERACRUZ | ON ATZOMPA XOXOCOTLA RD. 1.5 KMS FROM XOXOCOTLA | 18.6585 | -97.1574 | 1,524,182 | 2214 | 625.6 | 2479 | 16.0 | 82 | 13 | |
| MK 699 (1) | UMMZ 239791 | *Sarcohyla bistincta* | VERACRUZ | ON STREAM CROSSING THE TOWN OF XOXOCOTLA | 18.6477 | -97.1574 | 1,342,010 | 2206 | 610.9 | 1769 | 12.3 | 79 | 13 | |
| MK 700 (2) | UMMZ 239750 | *Sarcohyla bistincta* | PUEBLA | IN THE STREAM LOCATED AFTER THE TOWN OF ZOQUITLAN TURNING DOWN AT THE CENTRO DE SALUD | 18.322 | -97.0285 | 2,203,360 | 2298 | 697.8 | 4155 | 24.8 | 86 | 14 | |
| MK 705 (1) | UMMZ 239862 | *Sarcohyla bistincta* | OAXACA | 56 KMS FROM TEOTITLAN VIA HUAUTLA | 18.1576 | -96.8684 | 2,529,703 | 2417 | 644.6 | 3164 | 19.6 | 85 | 15 | |
| MK 715 | UMMZ 239755 | *Sarcohyla bistincta* | OAXACA | NEAR "EL TEJOCOTE" (FRENTE AL KINDER) | 17.239 | -97.0032 | 347,152 | 1712 | 437.7 | 254 | 4.3 | 31 | 16 | |
| MK 716 (1) | UMMZ 239758 | *Sarcohyla bistincta* | OAXACA | ON ROAD BETWEEN THE TOWNS OF SAN JUAN DEL ESTADO AND SAN MIGUEL ALOAPAN | 17.3036 | -96.793 | 52,461 | 594 | 267.4 | 8 | 3.0 | 1 | 17 | |
| MK 718 (2) | UMMZ 239765 | *Sarcohyla bistincta* | OAXACA | PASSING SAN MIGUEL ALOAPAN | 17.4211 | -96.6876 | 606,978 | 2014 | 568.6 | 366 | 4.9 | 37 | 18 | |
| MK 755 (1) | UMMZ 239786 | *Sarcohyla bistincta* | OAXACA | ON ROAD SAN JUAN ATEPEC SAN MIGUEL ABEJONES | 17.4153 | -96.5671 | 871,034 | 2062 | 563.2 | 1022 | 8.4 | 69 | 19 | |
| MK 751 | UMMZ 239785 | *Sarcohyla bistincta* | OAXACA | 3.8 KMS PASSING "RANCHO TEXAS" ON THE ROAD FROM THE TOWN OF IXTLAN DE JUAREZ | 17.316 | -96.4435 | 1,577,190 | 2342 | 621.5 | 2376 | 15.4 | 84 | 20 | MH899574 |
| MK 748 (2) | UMMZ 239780 | *Sarcohyla bistincta* | OAXACA | CA 37 KMS FROM MITLA ON THE ROAD MITLA AYUTLA | 16.9791 | -96.1364 | 91,912 | 1009 | 302.5 | 23 | 3.1 | 3 | 22 | |
| MK 748 (4) | UMMZ 239782 | *Sarcohyla bistincta* | OAXACA | CA 37 KMS FROM MITLA ON THE ROAD MITLA AYUTLA | 16.9791 | -96.1364 | 1,058,426 | 2170 | 553.8 | 499 | 5.7 | 42 | 22 | |
| MK 767 | UMMZ 239788 | *Sarcohyla bistincta* | OAXACA | 9.2 KMS E STA MARIA ALBARRADAS / SIERRA MIXE | 16.985888 | -96.135816 | 1,083,058 | 2206 | 524.7 | 617 | 6.3 | 52 | 22 | |
| MK 721 | UMMZ 239767 | *Sarcohyla bistincta* | OAXACA | 2 KMS N. THE TOWN OF STA. MARIA LAXICHIO VIA THE TOWN OF SAN SEBASTIAN RIO DULCE | 16.7377 | -97.0384 | 146,471 | 1215 | 376.0 | 62 | 3.3 | 10 | 23 | |
| MK 766 | UMMZ 239794 | *Sarcohyla bistincta* | OAXACA | CERRO DE VIDRIO VIA A PUERTO ESCONDIDO | 16.25216 | -97.15359 | 994,521 | 2166 | 577.4 | 467 | 5.5 | 43 | 24 | |
| MK 730 (2) | ? | *Sarcohyla bistincta* | ? | ? | | | 2,967,630 | 2404 | 716.9 | 3581 | 21.7 | 84 | | |
| MK 685 (2) | UMMZ 239739 | *Sarcohyla bistincta* | GUERRERO | ON ATOYAC PTO DE GALLO RD / BETWEEN 10 - 20 KMS NORTH OF THE TOWN "EL PARAISO" | 17.3812 | -100.2009 | 269,729 | 1645 | 450.6 | 184 | 4.0 | 20 | 25 | MH899575 |
| MK 689 (2) | UMMZ 239740 | *Sarcohyla bistincta* | GUERRERO | ON ATOYAC PTO DEL GALLO RD. / 500 M. NORTH OF THE TOWN OF SAN VICENTE | 17.3 | -100.2792 | 67,313 | 502 | 221.6 | 32 | 3.1 | 6 | 26 | |
| MK 727 (2) | UMMZ 239772 | *Sarcohyla pentheter* | OAXACA | RIO "EL SALADO" / 8 KMS N SAN JUAN LACHAO ON HWY 135 | 16.1916 | -97.0958 | 17,052 | 381 | 247.1 | 10 | 3.0 | 2 | 27 | MH899576 |
| MK 691 (3) | UMMZ 239651 | *Plectrohyla chryses* | GUERRERO | 2-3 KMS IN THE ROAD TO JALEACA FROM POINT WHERE ROAD TRIFURCATE TO PTO. DEL GALLO / YEXTLA / ANDJALEACA.COMMING FROM CARRIZAL DE BRAVO / IN RIVER UNDER THE BRIDGE | 17.5324 | -99.8994 | 2,267,946 | 2347 | 663.6 | 4919 | 33.4 | 83 | 28 | MH899570 |
| MK 770 | UMMZ 239802 | *Plectrohyla hazelae* | OAXACA | EL PUNTO SIERRA JUAREZ | 17.22156 | -96.58386 | 538,500 | 2066 | 526.2 | 557 | 5.9 | 44 | 29 | MH899569 |
| MK 667 | UMMZ 239952 | *Sarcohyla arborescandens* | VERACRUZ | PUERTO DEL AIRE (ARRIBA DE ALCUTZINGO) | 18.6787 | -97.3485 | 923,330 | 2116 | 511.8 | 1154 | 9.0 | 69 | | MH899576 |
| MK 700 (1) | UMMZ 239813 | *Sarcohyla cyclada* | PUEBLA | IN THE STREAM LOCATED AFTER THE TOWN OF ZOQUITLAN TURNING DOWN AT THE CENTRO DE SALUD | 18.322 | -97.0285 | 2,490,616 | 2385 | 661.4 | 2961 | 18.4 | 82 | | |
| MK 701 | UMMZ 239814 | *Sarcohyla cyclada* | OAXACA | 24 KMS FROM THE TOWN OF TEOTITLAN DE FLORES MAGON VIA HUAUTLA DE JIMENEZ | 18.1781 | -97.0054 | 1,166,574 | 2129 | 565.5 | 1061 | 8.7 | 69 | | |
| MK 742 (1) | UMMZ 239954 | *Sarcohyla arborescandens* | VERACRUZ | LEFT ROAD BIFURCATING FROM ROAD TO THE TOWN OF "LAS MINAS" | 19.6758 | -97.1751 | 1,074,225 | 2240 | 553.8 | 1750 | 12.0 | 79 | | |
| MK 768 | UMMZ 239833 | *Exerodonta xera* | PUEBLA | 5 KM SW ZAPOTITLAN DE SALINAS | 18.311958 | -97.51266 | 1,257,938 | 2288 | 581.5 | 3506 | 21.4 | 84 | | |

## Additional Files

Fig. S1. Sampled and unsampled parts of *S. bistincta* range in relation to known distributions (or localities, where distributional information is lacking) of other *Sarcohyla* species.

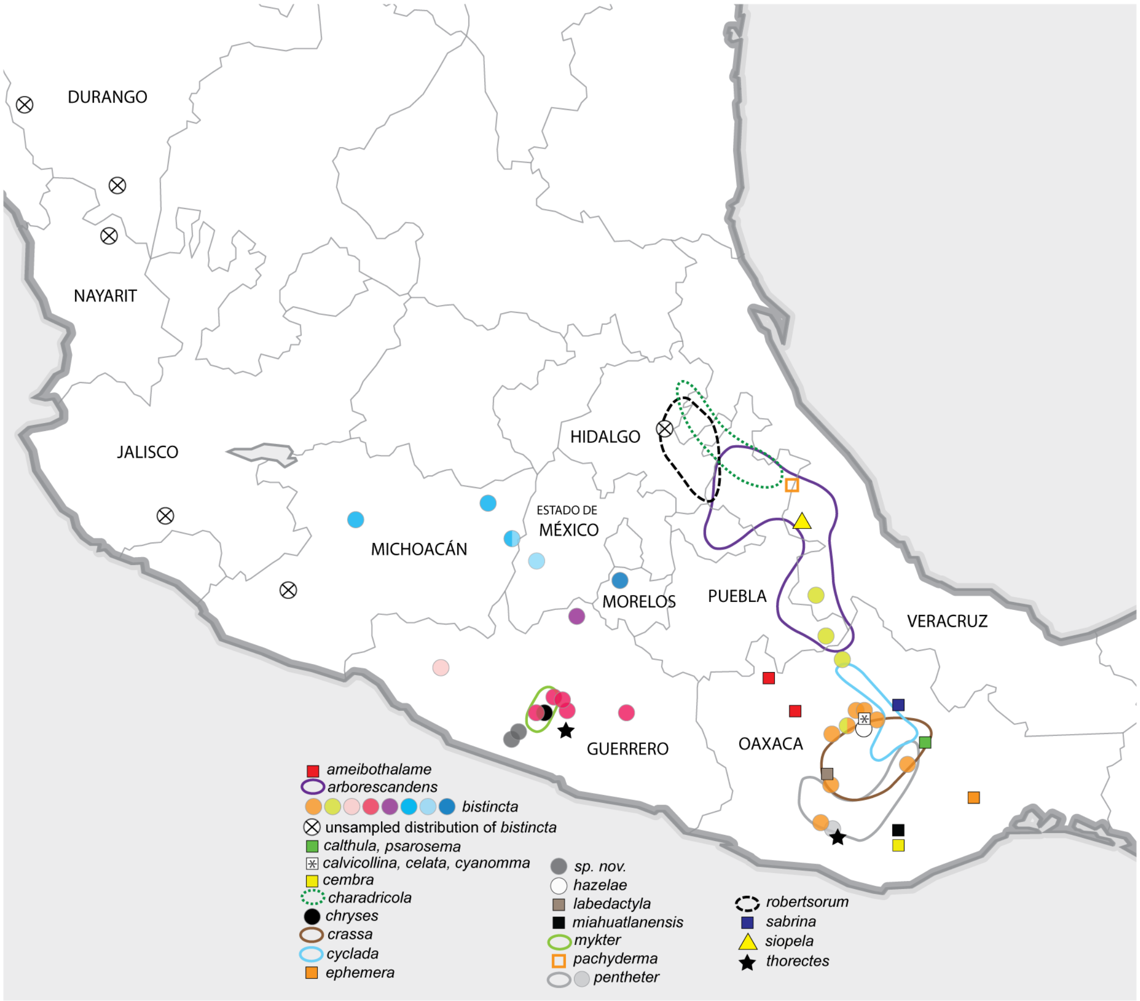

Fig. S2. UCE tree of 45 samples of *Sarcohyla* and outgroup *Exerodonta xera* used to determine the ingroup.

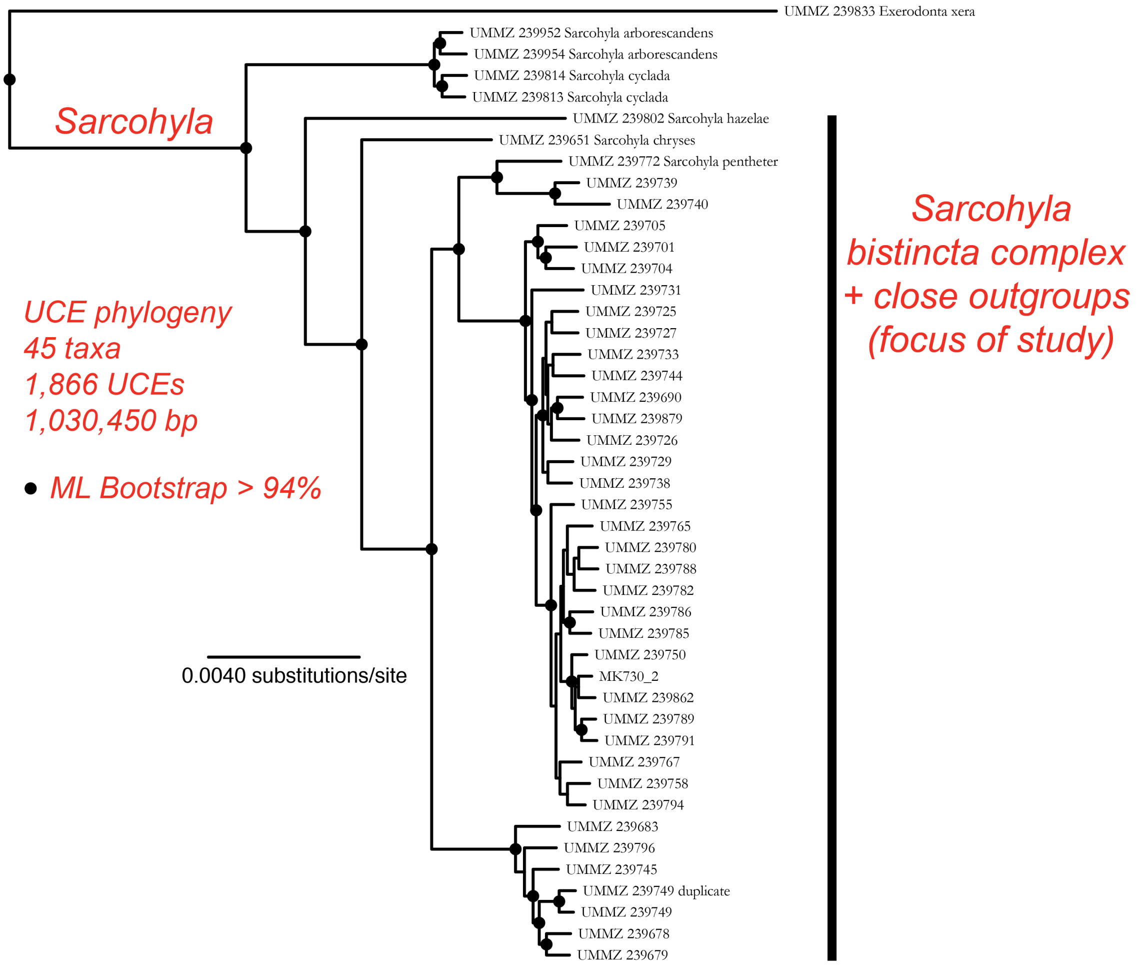

Supplement: Supplemental Information 1 — Table S1. Information and summary statistics on all 45 samples used to determine the ingroup for this study. Fig. S1. Sampled and unsampled parts of S. bistincta range in relation to known distributions (or localities, where distributional information is lacking) of other Sarcohyla species. Fig. S2. UCE tree of 45 samples of Sarcohyla and outgroup Exerodonta xera used to determine the ingroup. [file peerj-06-6045-s001.pdf]
